# The correlation between neck circumference and atherogenic index of plasma with coronary heart disease

**DOI:** 10.3389/fendo.2025.1562959

**Published:** 2025-10-03

**Authors:** Huihui Yang, Jie Dou, Ruoling Guo, Mingliang Sun, Jie Gao, Hongjun Shu, Hewei Sun, Xintao Zhao, Yuhua Song, Yanchun Hou, Ying Zhang, Donglei Luo

**Affiliations:** ^1^ Chengde Medical University Graduate School, Hebei, China; ^2^ Department of Cardiology, Chengde Central Hospital/Second Clinical College of Chengde Medical University, Hebei, China; ^3^ Department of Geriatrics, Chengde Central Hospital/Second Clinical College of Chengde Medical University, Hebei, China

**Keywords:** coronary heart disease, neck circumference, atherogenic index of plasma, severity of coronary lesions, restricted cubic spline

## Abstract

**Background and aims:**

Coronary heart disease (CHD) remains a leading cause of mortality globally and is becoming increasingly prevalent in China. Growing evidence suggests that dyslipidemia plays a critical role in the pathogenesis of atherosclerosis. The primary objective of this study was to investigate the association between neck circumference (NC) and the atherogenic index of plasma (AIP) with the severity of CHD, and to evaluate the diagnostic utility of combining NC with AIP for identifying CHD.

**Methods:**

This observational, cross-sectional study included 1652 patients with chest pain from the Department of Cardiology at Chengde Central Hospital who underwent coronary angiography (CAG). Restricted cubic splines (RCS) were used to examine the associations between NC, AIP, and CHD. Independent predictors of CHD risk were identified through univariate and multivariate regression analyses and subsequently integrated into a risk prediction nomogram. The model’s performance was validated using receiver operating characteristic (ROC) curve, calibration plots, and decision curve analysis (DCA).

**Results:**

The study demonstrates a robust association between NC and AIP with both the incidence and severity of CHD. After adjusting for sex and age, the ORs (95% CIs) for CHD increased progressively across the quartiles of NC: ORs were 1.24 (0.89-1.74), 1.42 (0.98-2.04), and 1.80 (1.22-2.66) for the second, third, and fourth quartiles, respectively, compared with the first quartile, indicating a significant increasing trend (*p-*trend = 0.030). The ORs for the severity of coronary lesions across higher NC quartile were 1.22 (0.89-1.65), 1.38 (1.00-1.90), and 1.41 (1.02-1.95), relative to the lowest quartile (*p-*trend = 0.039). The RCS curve demonstrated a significant positive linear relationship between NC, AIP, and CHD. The predictive accuracy of the nomogram model for CHD was substantial, evidenced by an area under the curve (AUC) of reaching 0.729 (95% CI 0.697-0.761, *p <*0.001).

**Conclusions:**

NC and AIP are positively correlate with CHD. Combined measurement of these variables provides significant predictive value for the diagnosis of CHD.

## 1 Introduction

Coronary heart disease (CHD) is a cardiovascular condition characterized by myocardial ischemia, hypoxia, and potential necrosis, which result from stenosis or complete occlusion of the coronary arteries due to lipid accumulation and calcification. CHD is a leading cause of global mortality, with a consistently rising incidence in China. The high rates of disability and mortality associated with CHD impose a substantial burden on both society and families. Despite significant advances in the diagnosis and treatment of CHD in recent decades, recent reports indicate that the prevalence of CHD in China has reached approximately 11.39 million individuals, with the mortality rate continuing to rise (1). Early detection and prevention of CHD have become focal points of research and clinical interest among Chinese experts and scholars. Well-established risk factors for CHD include advanced age, male sex, smoking, hypertension, and dyslipidemia. Among these, emerging evidence increasingly underscores dyslipidemia as a critical factor, acting as a key driver of both the generation and progression of CHD (2, 3).

Neck circumference (NC) is a significant anthropometric measure, reflecting lipid metabolism and the accumulation of subcutaneous fat in the upper body (4, 5). The pathophysiological mechanisms underlying the association between NC and CHD remain incompletely understood. Current evidence suggests that an increased NC, indicative of greater upper-body adiposity, may contribute to insulin resistance and vascular dysfunction through the excessive release of free fatty acids. This process potentially promotes endothelial dysfunction and enhances the synthesis of low-density lipoprotein cholesterol (LDL-C) (6). In the early stages of obesity, the accumulation of subcutaneous adipose tissue results in both hypertrophy and hyperplasia of adipocytes, predominantly affecting preadipocytes. As adipocyte hypertrophy progresses, the storage capacity of subcutaneous fat is diminished, leading to a pathological redistribution of fat to visceral depots. This process contributes to the development of central obesity, thereby increasing the risk of CHD (7). Additionally, the deposition of fat around blood vessels (perivascular fat) and within the myocardium (perimyocardial fat) may exacerbate hypertension and vascular stiffness, further promoting the pathogenesis of CHD (8). In contrast to conventional anthropometric measurements, such as waist and abdominal circumference, NC has demonstrated a significant correlation with cardiovascular risk factors and insulin resistance. It offers several advantages, including ease of measurement, accessibility, low cost, and high reproducibility, making it as a promising external marker for the assessment of CHD (9, 10).

In recent years, novel biomarkers derived from routine blood tests have been increasingly investigated for their potential in the early diagnosis of CHD, risk stratification, and guiding prevention and therapeutic strategies. The atherogenic index of plasma (AIP), a recently proposed lipid metric, is calculated as logarithm of the ratio of plasma triglyceride (TG) to plasma high-density lipoprotein cholesterol (HDL-C). Emerging evidence indicates that AIP serves as an independent predictor of CHD, with superior predictive performance compared with conventional lipid parameters (11, 12). Rooted in the hypothesis that lipid metabolism dysregulation plays a central role in the initiation and progression of CHD, recent clinical studies have consistently identified both NC and AIP as robust predictors of CHD occurrence (9, 11). However, the existing literature remains limited in its exploration of NC and AIP in assessing the severity of CHD in affected individuals. This study aims to evaluate the relationships between NC and AIP levels with respect to both CHD incidence and lesion severity. Furthermore, we seek to assess the diagnostic value of combining these parameters for the detection and risk stratification of CHD, thereby providing a theoretical foundation for the early identification of the disease and the stratification of patient risk.

## 2 Patients and methods

### 2.1 Study population and ethics

**Figure 1:**
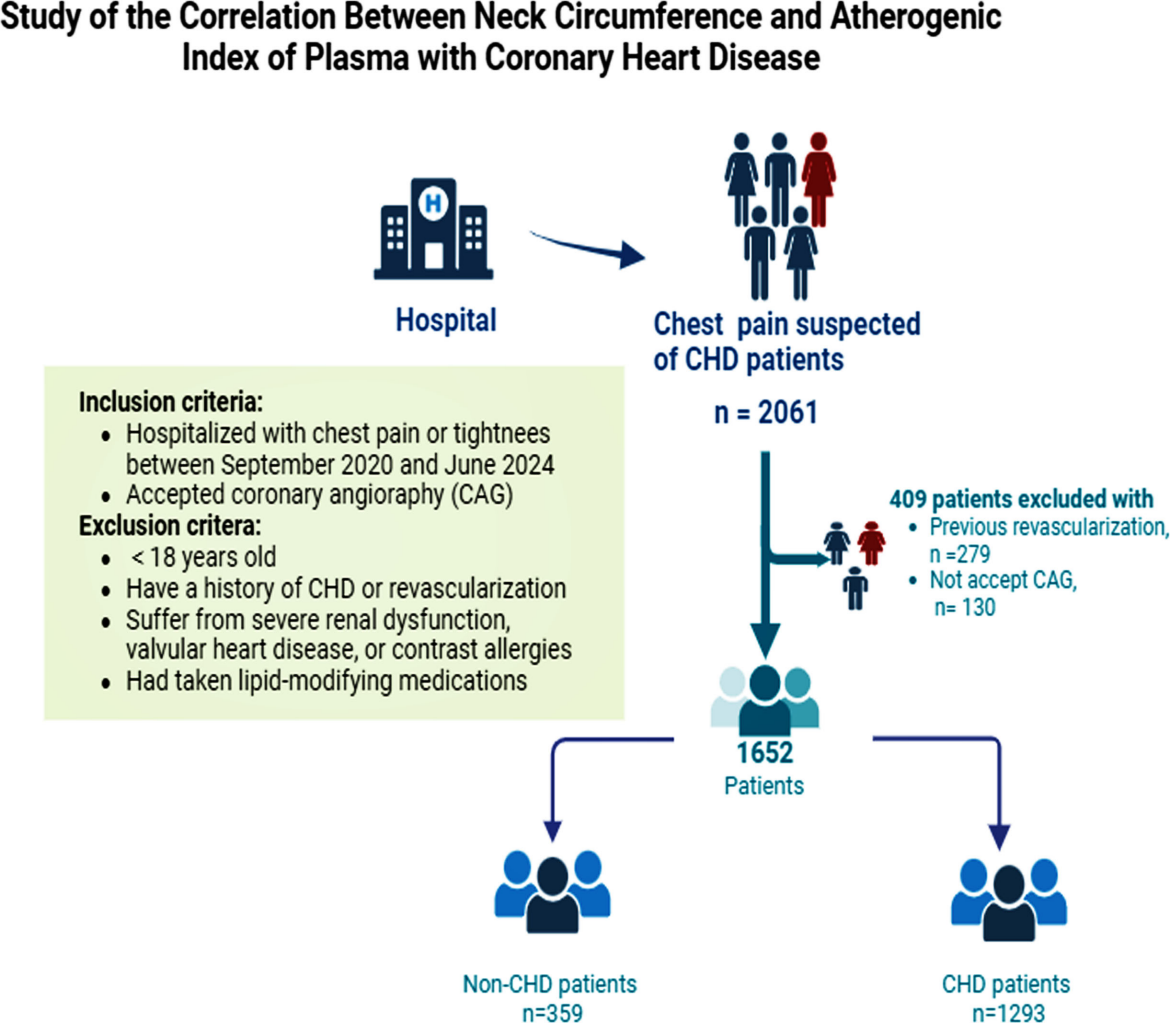
A detailed flow chart of participant recruitment. CHD, coronary heart disease; CAG, coronary angiography.

This single-center, cross-sectional study was conducted in accordance with the Declaration of Helsinki and received approval from the Ethics Committee of Chengde Central Hospital. Written informed consent was obtained from all participants. 2061 patients with chest pain and suspected CHD were hospitalized in the Department of Cardiovascular Medicine at Chengde Central Hospital from September 2020 to June 2024. We excluded 279 patients with a history of prior revascularization and 130 patients who did not undergo coronary angiography (CAG). A total of 1652 patients were included in the final statistical analysis to investigate the association between NC, AIP, and CHD. Patients were excluded based on the following criteria: (1) age <18 years; (2) a history of CHD or revascularization; (3) severe renal dysfunction, valvular heart disease, or contrast allergies; (4) recent use of lipid-lowering medications; and (5) failure to undergo CAG. This study is registered with the Chinese Clinical Trial Registry, identifier ChiCTR2000041499, and is available at http://www.chictr.org.cn. A detailed flow chart of the study design is provided in **Figure 1**.

### 2.2 Data collection and definition

#### 2.2.1 Measurement of NC

NC was measured with the subject in an upright position, with the eyes directed forward. A flexible tape measure was placed horizontally at the upper margin of the laryngeal prominence. Measurements were performed twice by trained clinicians or medical students. The precision of the measurement was 0.1cm, and the difference between the two readings was required to be less than 0.5cm. The mean of the two measurements was used for analysis.

#### 2.2.2 General baseline data and laboratory indicators

Guided by existing literature and biological considerations, we collect a comprehensive set of covariates known to influence CHD outcomes. The baseline data included age, sex, medical history (hypertension, diabetes, hyperuricemia, stroke), personal history (smoking and alcohol consumption), and a detailed family history of CHD and diabetes. Upon admission, we meticulously recorded the patient’s anthropometric and hemodynamic parameters, including height, weight, heart rate, and blood pressure, with specific measurements of systolic blood pressure (SBP) and diastolic blood pressure (DBP). Body mass index (BMI) was calculated using the standard formula: weight (kg)/square of height (m^2^). Serum levels of alanine aminotransferase (ALT), aspartate aminotransferase (AST), triglyceride (TG), total cholesterol (TC), high-density lipoprotein cholesterol (HDL-C), and low-density lipoprotein cholesterol (LDL-C) were quantified through rigorous, standardized laboratory procedures. All participants underwent resting electrocardiograms and echocardiograms. Physical examinations and laboratory tests were conducted by a skilled team of medical practitioners. The atherogenic index of plasma (AIP) was calculated as the logarithm of (TG/HDL-C), expressed as AIP = log_10_(TG/HDL-C) (12).

#### 2.2.3 Relevant diagnostic criteria and definition

CHD, as defined by the European Society of Cardiology, is characterized by a luminal narrowing of at least 50% in one or more coronary arteries or their major branches, as assessed by CAG (13). Hypertension is clinically diagnosed based on repeated office-based measurements showing a SBP ≥ 140 mmHg and/or DBP ≥ 90 mmHg, recorded on at least three separate occasions without the use of antihypertensive medications. Furthermore, individuals with a previous diagnosis of hypertension maintain their classification as hypertensive, even if blood pressure measurements fall below the 140/90 mmHg threshold due to the sustained use of antihypertensive therapy (14). Diabetes is diagnosed according to the criteria established by the World Health Organization (WHO) Diabetes Mellitus Expert Committee (15). Hyperlipidemia is diagnosed in accordance with the latest lipid management guidelines in China (16). Hyperuricemia in males is diagnosed when fasting serum uric acid levels exceed 420 μmol/L on two separate occasions while adhering to a normal purine diet. In females, the diagnostic threshold is 360 μmol/L (17). ST-T changes are diagnosed based on the following criteria: ST segment depression exceeding 0.05 millivolts, T wave inversions, T wave amplitudes decreasing to less than 1/10 of the R wave amplitude, and the subsequent normalization of the electrocardiogram following symptomatic relief (18). According to the WHO, smoking is defined as the habitual consumption of at least one cigarette daily for a period exceeding six months, irrespective of whether the individual has since quit (19).

### 2.3 Coronary angiography and Gensini score calculation

All enrolled participants underwent CAG, and the angiographic results were independently assessed by at least two experienced interventional cardiologists. In the event of discrepancies, a third cardiologist was consulted to reach a consensus. The Gensini score is a widely employed angiographic scoring system for assessing the severity of coronary artery disease, determined according to the ACC/AHA guidelines for coronary vessel scoring, as detailed in **Table 1** (20). In this study, the Gensini score was independently calculated by two cardiologists who had received standardized training. In cases of discrepancies, re-evaluation and recalculation were conducted to achieve consensus.

**Table 1:** Gensini score standard.

Degree of stenosis	Score	Coronary artery lesion site	Coefficient
1 - 25%	1	Left Main	×5
26 - 50%	2	LAD proximal segment, LCX proximal segment	×2.5
51 - 75%	4	LAD mid-segment	×1.5
76 - 90%	8	RCA proximal segment, RCA mid-segment, RCA distal segment, PDA, LAD distal segment, 1^st^ Diagonal	×1
91 - 99%	16	other segments	×0.5
100%	32	Obtuse Marginal, LCX mid-segment, LCX distal segment	×1

Gensini score was calculated by summation of the individual coronary segment scores.

LAD, left anterior descending coronary artery; LCX, left circumflex artery; RCA, right coronary artery; PDA, posterior descending artery.

### 2.4 Statistical analysis

Statistical analyses were performed using SPSS version 26.0 and R version 4.4.0. Initially, the normality of all continuous variables was assessed. For continuous variables following a normal distribution, data are presented as mean ± standard deviation, and inter-group comparisons were conducted using the independent two-sample Student’s t-test. For variables not normally distributed, data are summarized as median (*p*25, *p*75), and inter-group comparisons were performed using the Mann-Whitney U test. Categorical variables are presented as counts and percentages (%), with the Chi-square test employed to assess inter-group differences. Participants were stratified into quartiles based on NC as follows: Q1 group, NC < 35.50cm; Q2 group, 35.50cm ≤ NC < 38.00cm; Q3 group, 38.00cm ≤ NC< 40.00cm; and Q4 group, NC ≥ 40.00cm, with the first quartile (Q1) serving as the reference group. Patients with CHD were classified into a mild disease group (GS ≤ 18 points) and a severe disease group (GS > 18 points) based on the median score.

We employed multivariate logistic regression to ascertain the association between NC and CHD, as well as the severity of coronary lesions, including both the unadjusted model and the adjusted model (Model I). Odds ratios (ORs) and their respective 95% confidence intervals (CIs) were calculated to assess the relationship between NC and CHD, as well as the impact of NC on the severity of coronary lesions. Adjustments for age and sex were incorporated in Model I. To further investigate the potential nonlinearity of the relationship between NC, the AIP, and CHD, cubic spline regression (RCS) was applied. Based on a review of the relevant literature and considering model fit and the smoothness of the RCS curve, a logistic regression model with four knots, positioned at the 5th, 35th, 65th, and 95th percentiles, was selected (21, 22).

To identify the most critical predictors of CHD among various variables, we employed both univariate and multivariate logistic regression models. A predictive nomogram for CHD risk was then constructed, integrating several critical CHD-associated variables. The discriminatory ability of this nomogram to predict CHD risk was validated using the receiver operating characteristic (ROC) curve. To evaluate the clinical validity of the diagnostic method, we applied a comprehensive approach that included decision curve analysis (DCA) and the construction of a clinical impact curve (CIC). The potential for multicollinearity among covariates was reviewed by the Variance Inflation Factor (VIF), with a VIF threshold of > 3 indicative of significant collinearity (23). Statistical significance was defined as a *p*-value < 0.05, determined using a two-tailed test.

## 3 Results

### 3.1 Baseline characteristics of the study participants

A total of 1,652 participants were included in the analysis, with a mean age of 60.33 years. The mean NC among all participants was 37.85cm, while the mean AIP was 0.18. The results revealed that individuals with CHD had significantly higher levels of NC (38.03 vs. 37.02cm, *p*<0.001) and AIP (0.19 vs. 0.13, *p*<0.001) compared to those without CHD. Furthermore, CHD patients were more likely to be older, male, smokers, and drinkers, as well as to have diabetes and hypertension. They also exhibited higher rates of wall-motion abnormalities and ST-T change. Additionally, CHD patients had higher levels of Gensini score, SBP, AST, TG, and LDL-C, accompanied by a reduction in HDL-C (*p*<0.05) (**Table 2**).

**Table 2:** Baseline characteristics of the study population grouped by CHD status.

Variable	Total (n = 1652)	CHD (n=1293)	Non-CHD (n=359)	*t*/*χ^2^*/*Z*	*p-*value
Age (years)	60.33 ± 9.48	60.80 ± 9.55	58.66 ± 9.03	-3.787	< 0.001
Sex (%)				37.669	< 0.001
Male	965(58.40)	806(62.30)	159(44.30)		
Female	687(41.60)	487(37.70)	200(55.70)		
BMI (kg/m^2^)	25.57 ± 3.47	25.57 ± 3.49	25.58 ± 3.38	0.052	0.958
NC (cm)	37.85 ± 3.24	38.03 ± 3.22	37.02 ± 3.19	-5.523	< 0.001
Heart rate (beats/min)	76.60 ± 12.87	76.44 ± 13.05	77.15 ± 12.18	0.929	0.353
SBP (mmHg)	137.03 ± 20.29	137.78 ± 20.65	134.34 ± 18.71	-2.846	0.003
DBP (mmHg)	82.56 ± 12.70	82.55 ± 13.04	82.60 ± 11.44	0.063	0.949
Smoking (%)	764(46.20)	643(49.70)	121(33.70)	29.025	< 0.001
Drinking (%)	553(33.50)	450(34.80)	103(28.70)	4.714	0.030
Previous history					
Hypertension (%)	1030(62.30)	842(65.10)	188(52.40)	19.465	< 0.001
Diabetes (%)	466(28.20)	399(30.90)	67(18.70)	20.636	< 0.001
Hyperuricemia (%)	306(18.50)	232(17.90)	74(20.60)	1.327	0.249
Stroke (%)	288(19.20)	241(16.10)	47(14.20)	6.957	0.008
Family history of CHD (%)	167(10.10)	135(10.40)	32(8.90)	0.721	0.396
Family history of diabetes (%)	59(3.60)	48(3.70)	11(3.10)	0.343	0.558
Laboratory test index					
ALT	21.00(16.00, 31.00)	21.00(16.00, 31.00)	20.00(15.00, 30.00)	-1.458	0.145
AST	20.00(16.00, 27.00)	20.00(17.00, 28.00)	19.00(16.00, 25.00)	-3.190	0.001
TG (mmol/L)	1.59(1.12, 2.32)	1.61(1.12, 2.41)	1.53(1.13, 2.08)	-2.068	0.039
TC (mmol/L)	4.29 ± 1.07	4.30 ± 1.09	4.23 ± 0.99	-1.148	0.251
HDL-C (mmol/L)	1.08(0.93, 1.28)	1.07(0.92, 1.25)	1.16(0.99, 1.37)	-5.356	< 0.001
LDL-C (mmol/L)	2.33 ± 0.82	2.33 ± 0.83	2.25 ± 0.76	-2.05	0.041
AIP	0.18 ± 0.30	0.19 ± 0.31	0.13 ± 0.27	-4.120	< 0.001
Wall-motion abnormalities (%)	951(57.60)	787(60.90)	164(45.70)	25.519	< 0.001
ST-T change (%)	841(50.90)	722(55.80)	119(33.10)	57.891	< 0.001
Gensini	10.00(2.00, 32.00)	18.00(6.00, 41.00)	0.00(0.00, 00.00)	-28.293	< 0.001

CHD, coronary heart disease; BMI, body mass index; NC, neck circumference; SBP, systolic blood pressure; DBP, diastolic blood pressure; ALT, alanine aminotransferase; AST, aspartate aminotransferase; TG, triglyceride; TC, total cholesterol; HDL-C, high-density lipoprotein cholesterol; LDL-C, low-density lipoprotein cholesterol; AIP, atherogenic index of plasma.

### 3.2 Analysis of baseline characteristics among different severity groups of CHD

In the comparison between mild and severe disease groups, patients with severe disease demonstrated a significantly higher NC than those with mild disease (38.32 vs. 37.84cm, *p*=0.008). Additionally, the AIP was markedly higher in the severe disease cohort compared to the mild disease group (0.22 vs. 0.17, *p*=0.002). Patients with severe disease were more likely to be male, smokers, and to have diabetes than those with mild disease. They also exhibited higher prevalence of wall-motion abnormalities and ST-T changes. Furthermore, patients with severe disease had elevated levels of ALT, AST, TG, and LDL-C, with a concurrent decrease in HDL-C (*p*<0.05) (**Table 3**).

**Table 3:** Baseline characteristics of the CHD patients grouped by the severity of coronary artery disease.

Variable	Mild disease group (n=632)	Severe disease group (n=661)	*t*/*χ^2^*/*Z*	*p-*value
Age (years)	60.81 ± 9.04	60.78 ± 10.05	0.047	0.963
Sex (%)			14.323	< 0.001
Male	361(57.10)	445(67.30)		
Female	271(42.90)	216(32.7)		
BMI (kg/m^2^)	25.76 ± 3.50	25.40 ± 3.48	1.859	0.063
NC (cm)	37.84 ± 3.24	38.32 ± 3.19	-2.648	0.008
Heart rate (beats/min)	76.63 ± 13.03	76.25 ± 13.09	0.523	0.601
SBP (mmHg)	138.51 ± 19.68	137.08 ± 21.54	1.247	0.213
DBP (mmHg)	82.95 ± 12.30	82.17 ± 13.71	1.072	0.284
Smoking (%)	277(43.80)	366(55.40)	17.216	< 0.001
Drinking (%)	210(33.20)	240(36.30)	1.351	0.245
Previous history				
Hypertension (%)	399(63.10)	443(67.00)	2.149	0.143
Diabetes (%)	154(24.40)	245(37.10)	24.416	< 0.001
Hyperuricemia (%)	112(17.70)	120(18.20)	0.041	0.839
Family history of CHD (%)	63(10.00)	72(10.90)	0.295	0.587
Family history of diabetes (%)	21(3.30)	27(4.10)	0.525	0.469
Laboratory test index				
ALT	21.00(15.00, 30.00)	22.00(16.00, 33.00)	-2.063	0.039
AST	20.00(16.00, 25.00)	21.00(17.00, 32.00)	-4.184	< 0.001
TG (mmol/L)	1.55(1.09, 2.30)	1.68(1.14, 2.48)	-2.091	0.037
TC (mmol/L)	4.26 ± 1.10	4.36 ± 1.08	-1.678	0.092
HDL-C (mmol/L)	1.10(0.94, 1.30)	1.04(0.89, 1.21)	-4.436	< 0.001
LDL-C (mmol/L)	2.29 ± 0.82	2.40 ± 0.84	-1.2.585	0.010
AIP	0.17 ± 0.32	0.22 ± 0.30	-3.162	0.002
Wall-motion abnormalities (%)	353(55.90)	434(65.70)	13.037	< 0.001
ST-T change (%)	292(46.20)	430(65.10)	46.557	< 0.001

CHD, coronary heart disease; BMI, body mass index; NC, neck circumference; SBP, systolic blood pressure; DBP, diastolic blood pressure; ALT, alanine aminotransferase; AST, aspartate aminotransferase; TG, triglyceride; TC, total cholesterol; HDL-C, high-density lipoprotein cholesterol; LDL-C, low-density lipoprotein cholesterol; AIP, atherogenic index of plasma.

### 3.3 Association of NC with CHD

**Figure 2:**
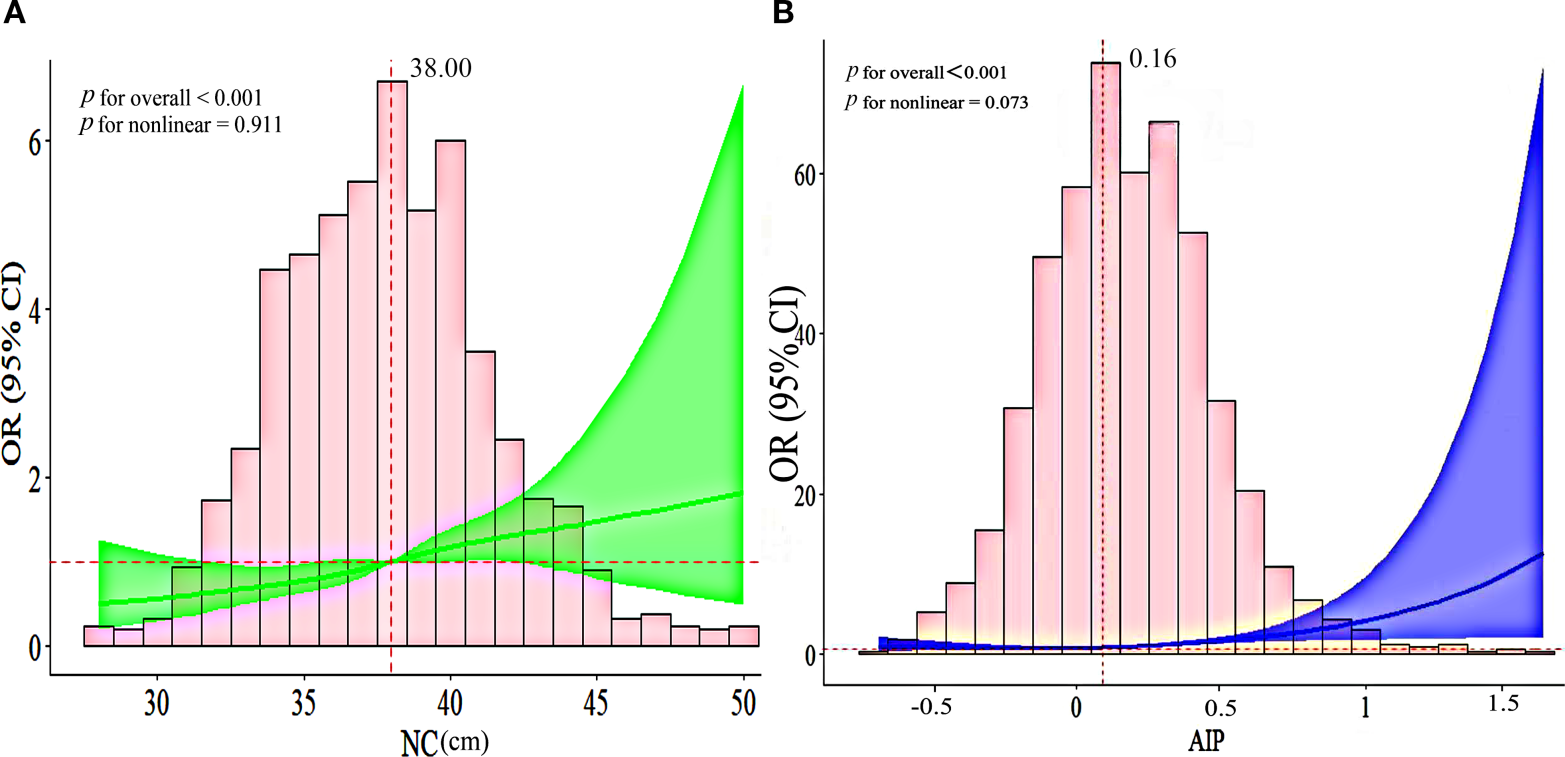
**(A)** The RCS curve of the association between NC and CHD among all the study participants; **(B)** The RCS curve of the association between AIP and CHD among all the study participants. OR, odds ratio; CI, confidence interval; NC, neck circumference; AIP, atherogenic index of plasma.

The multivariate logistic regression analysis, as presented in **Table 4**, was conducted to assess the association between NC and CHD. Our results indicate that an increase in NC is significantly associated with an elevated risk of CHD. After adjusting for potential confounders, the ORs (95% CIs) for CHD progressively increased across the quartiles of NC. The ORs of the second, third, and fourth quartiles were 1.24 (0.89-1.74), 1.42 (0.98-2.04), and 1.80 (1.22-2.66), respectively, compared to the first quartile, indicating a significant increasing trend (*p-*trend = 0.030). The RCS curve, shown in **Figure 2**, reveals a positive linear relationship between NC and CHD. Notably, an NC value exceeding 38.00cm is associated with a significant increase in CHD risk. Similarly, an AIP value greater than 0.16 is correlated with a gradual elevation in CHD risk.

**Table 4:** Logistic regression analysis on the association between NC and CHD.

Variable	Non-adjusted model			Model I		
	OR	95% CI	*p-*value	OR	95% CI	*p-*value
NC	1.11	1.07-1.15	< 0.001	1.07	1.03-1.12	0.002
Q1	Reference			Reference		
Q2	1.48	1.08-2.04	0.016	1.24	0.89-1.74	0.201
Q3	1.81	1.30-2.53	< 0.001	1.42	0.98-2.04	0.064
Q4	2.41	1.74-3.33	< 0.001	1.80	1.22-2.66	0.003
*P* for trend			< 0.001			0.003

Data are presented as OR and 95% CI. Model I was adjusted for age, sex. OR, odds ratio; CI, confidence interval; NC, neck circumference; CHD, coronary heart disease; Q1, 1st quartile; Q2, 2nd quartile; Q3, 3rd quartile; Q4, 4th quartile.

### 3.4 Identification of critical predictors of CHD

**Figure 3:**
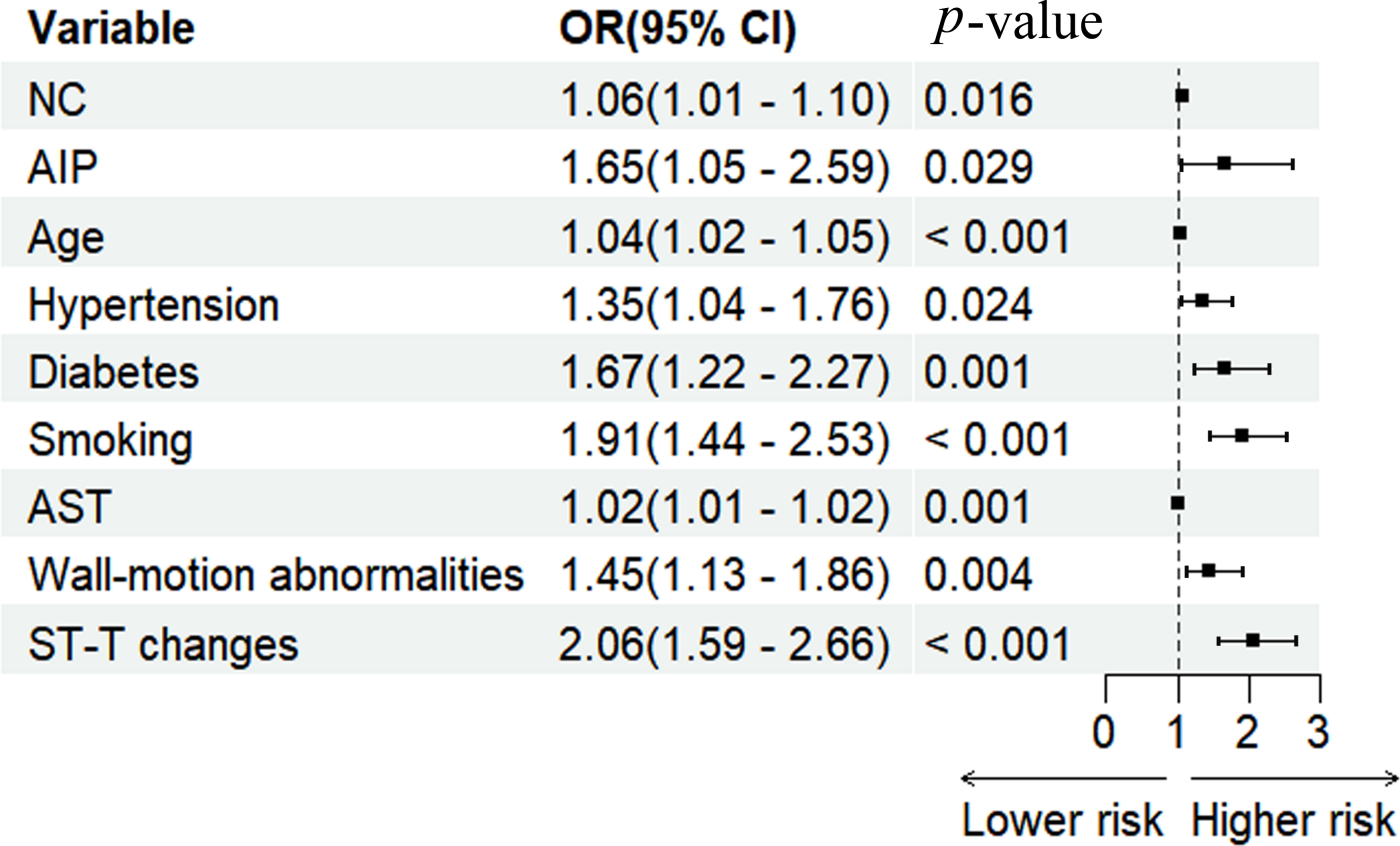
Forest plot for multivariate regression analysis of the participant. NC, neck circumference; AIP, atherogenic index of plasma; AST, aspartate aminotransferase; OR, odds ratio; CI, confidence interval.

**Figure 4:**
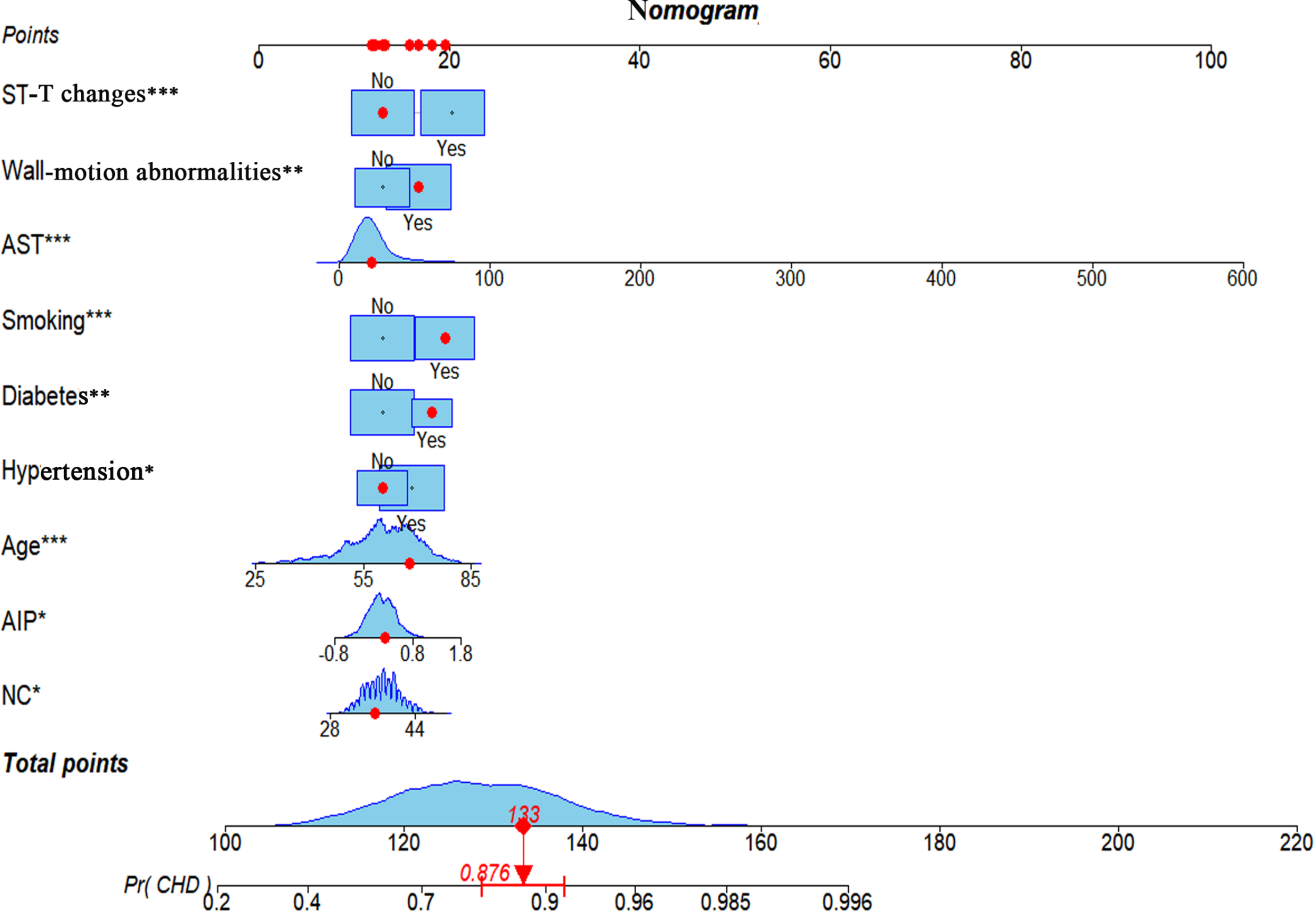
A nomogram model based on NC, AIP, age, hypertension, diabetes, smoking, AST, wall-motion abnormalities, and ST-T changes identified by multivariate regression analysis. * *p*-value < 0.05, ** *p*-value < 0.01, *** *p*-value ≤ 0.001. NC, neck circumference; AIP, atherogenic index of plasma; AST, aspartate aminotransferase; CHD, coronary heart disease.

A risk prediction model was constructed utilizing multivariate logistic regression analysis. The results identified NC, AIP, age, hypertension, diabetes, smoking, AST, wall-motion abnormalities, and ST-T changes as independent predictors of CHD. To assess the potential for multicollinearity, the VIF test was employed, revealing that all variables had VIF values < 2, indicating the absence of multicollinearity. A forest plot based on these results was generated to represent the strength and direction of these associations visually, and the detailed consequences are provided in **Table 5** and graphically described in **Figure 3**. A nomogram-based risk prediction model was then constructed, integrating all nine aforementioned variables (**Figure 4**).

**Table 5:** Univariate and multivariate logistic regression analysis of the characteristics of the CHD.

Variable	Univariate logistic regression analysis			Multivariate logistic regression analysis		
	OR	95% CI	*p-*value	OR	95% CI	*p-*value
NC	1.110	1.069-1.153	< 0.001	1.056	1.010-1.104	0.016
AIP	2.172	1.452-3.249	< 0.001	1.651	1.053-2.588	0.029
Age	1.023	1.011-1.036	< 0.001	1.038	1.023-1.053	< 0.001
Hypertension	1.698	1.340-2.151	< 0.001	1.355	1.041-1.762	0.024
Diabetes	1.945	1.455-2.601	< 0.001	1.666	1.220-2.275	0.001
Smoking	1.946	1.524-2.485	< 0.001	1.906	1.438-2.526	< 0.001
AST	1.021	1.011-1.030	< 0.001	1.016	1.006-1.025	0.001
Wall-motion abnormalities	1.849	1.461-2.341	< 0.001	1.450	1.128-1.864	0.004
ST-T changes	2.550	1.995-3.260	< 0.001	2.055	1.586-2.663	< 0.001

NC, neck circumference; AIP, atherogenic index of plasma; AST, aspartate aminotransferase; CHD, coronary heart disease; OR, odds ratio; CI, confidence interval.

### 3.5 Predictive model validation

**Figure 5:**
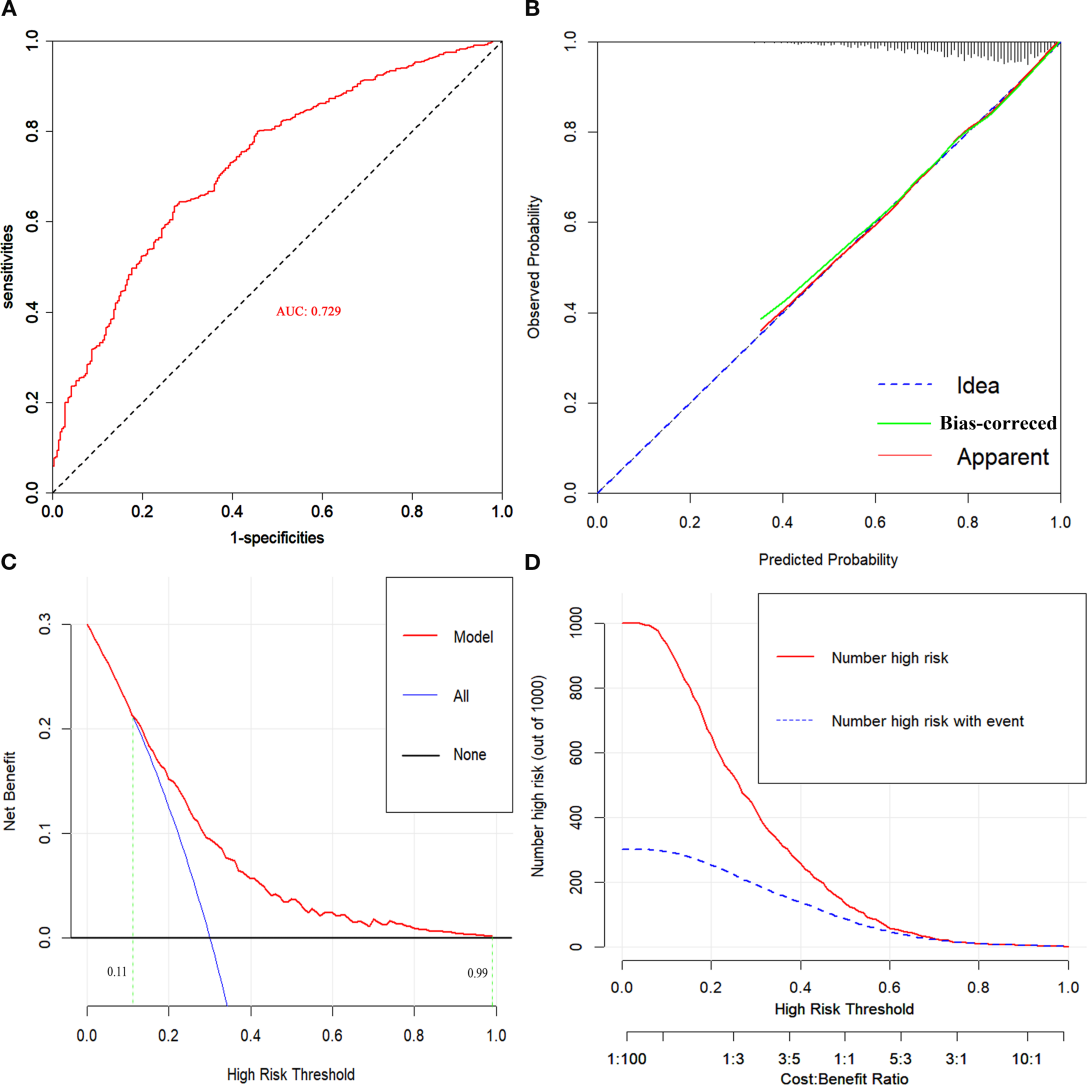
**(A)** ROC curve for evaluating the predictive power for CHD of the nomogram model. **(B)** Calibration plot for the nomogram model. **(C)** DCA curves for the nomogram model. **(D)** CIC for the nomogram model. AUC, area under the receiver operating characteristic curve.

The discriminative ability of the nomogram was assessed using ROC analysis, which yielded an AUC of 0.729 (95% CI: 0.697-0.761), demonstrating strong predictive accuracy for CHD (**Figure 5A**). To evaluate the goodness of fit, calibration curves were constructed, showing excellent concordance between predicted probabilities and the observed incidence of CHD. The Hosmer-Lemeshow test for the nomogram was *χ*2 = 4.579 (*p*=0.802), indicating a reliable model fit (**Figure 5B**). DCA and CIC were conducted to assess the clinical utility of the risk-stratification model in clinical settings. The DCA curves indicated that the nomogram provides predictive benefits for CHD across a broad threshold probability ranging from 11 to 99%, with the results graphically represented in **Figures 5C, D**.

### 3.6 Comparison of differences between neck circumference and waist circumference for coronary heart disease

In the multivariate regression model, adjusted for age and gender, the odds ratio (OR) for NC was 1.08 (95% CI: 1.03-1.12, *p*=0.002), which was higher than that for waist circumference (WC) (OR=1.00, 95% CI: 0.99-1.02, *p*=0.598) (**Table 6**). ROC curve analysis revealed that the AUC for NC was 0.596, compared with 0.520 for WC. The Delong test indicated a statistically significant difference between the two indicators (Z=4.645, *p*<0.001) (**Table 7**).

**Table 6:** Comparison of the strength of association between neck circumference, waist circumference, and CHD.

Variable	OR	95% CI	*p-*value
NC	1.08	1.03-1.12	0.002
WC	1.00	0.99-1.02	0.598

Adjusted for age and gender.

NC, neck circumference; WC, waist circumference.

**Table 7:** Comparison of the discriminative abilities of neck circumference and waist circumference for CHD.

Variable	AUC	95% CI	*p-*value
NC	0.596	0.562-0.629	< 0.001
WC	0.520	0.487-0.554	0.231

Adjusted for age and gender.

NC, neck circumference; WC, waist circumference.

## 4 Discussion

CHD remains one of the leading global causes of mortality, posing a major threat to public health and severely impairing quality of life worldwide (24). Early detection, prevention, and timely intervention are crucial for improving patient prognosis. Currently, CAG remains the gold standard for diagnosing CHD, providing direct visualization of coronary artery stenosis (25). However, this invasive diagnostic modality is associated with limitations, including high costs and complex technical demands. As such, there is a pressing need to develop non-invasive, widely accessible, and repeatable diagnostic methods for the early identification of CHD. Therefore, this study seeks to investigate the associations between NC, AIP, and the incidence and severity of CHD. Additionally, it aims to evaluate the diagnostic performance of a combined approach utilizing NC and AIP in the identification of CHD, thereby offering a theoretical basis for early diagnosis and risk stratification based on these parameters.

In our study of 1652 participants, we observed that individuals with CHD had significantly higher mean NC and AIP compared to those without CHD. Additionally, we found a strong association between elevated NC and an increased risk of severe coronary artery lesions. Our study provides the first confirmation of a linear and positive correlation among NC, AIP, and CHD through the application of RCS. Multivariate regression analysis revealed that both NC and AIP are independent risk factors for CHD. By integrating these critical CHD-associated variables, including age, hypertension, diabetes, smoking, AST, wall-motion abnormalities, and ST-T changes, we constructed and validated a nomogram model that demonstrates robust predictive accuracy for CHD.

Atherosclerosis (AS) is widely recognized as the primary pathophysiological driver of cardiovascular diseases, with dyslipidemia identified as a major risk factor for AS. Dyslipidemia is commonly associated with AS and is characterized by elevated levels of small and dense low-density lipoprotein (sdLDL), decreased HDL-C, and increased TG. However, studies have demonstrated that traditional single lipid indices do not fully capture the complexities of abnormal lipid metabolism (26). Notably, even when LDL-C is maintained within recommended thresholds, the residual risk of cardiovascular disease remains as high as 50% (27). Consequently, it is imperative to identify novel biomarkers that are both safe and cost-effective for the prompt recognition of individuals at elevated risk for CHD. Recent evidence suggests that NC has emerged as a pivotal anthropometric measure associated with lipid metabolism and the distribution of upper body subcutaneous fat (5). Several studies have highlighted the relationship between NC and cardiovascular risk factors and insulin resistance, emphasizing that NC could serve as a reliable external marker for the early identification of CHD. Unlike other indices, such as BMI, waist-hip ratio, and waist circumference, NC is impervious to factors such as eating, posture, and breathing, offering distinct advantages, including simplicity of measurement, cost-effectiveness, and reproducibility, thereby establishing it as a crucial tool in both clinical practice and research (9, 10). Furthermore, some researchers have suggested that the logarithmic ratio of TG to HDL-C closely correlates with sdLDL, providing a more precise reflection of lipid metabolism disorders. This metric, known as the AIP, has been shown to provide superior predictive value for cardiovascular diseases (28).

NC is recognized as a pivotal marker for assessing upper body subcutaneous fat. Its clinical significance is considered to surpass that of visceral fat (29) and has shown a strong correlation with cardiometabolic syndrome (30). First, NC measurement is characterized by its simplicity, non-invasiveness, and high acceptability. Unlike waist and hip circumference measurements, which may require extensive clothing removal and could cause discomfort in certain cultural contexts, NC can be easily measured without substantial disruption, thereby enhancing its feasibility for large-scale population screening and epidemiological studies. Second, waist and hip circumferences are subject to significant variability influenced by various factors, including meal intake, abdominal distension, and respiratory fluctuations (inhalation and exhalation), which can result in fluctuations of several centimeters within a single day. In contrast, NC, composed of both osseous and soft tissue structures, exhibits greater stability and is less affected by factors such as food consumption, respiratory phase, intestinal gas, or bladder distention. NC measurement is based on the identification of the thyroid cartilage (Adam’s apple) and superior margin of the seventh cervical vertebra, yielding highly reproducible measurements with minimal variation across different time points. Third, NC measurement offers particular advantages in specific populations. For individuals with severe obesity, obtaining accurate waist and hip circumference measurements can be technically challenging due to factors such as insufficient tape length or difficulty identifying bony landmarks like the anterior superior iliac spine. In contrast, measuring NC remains straightforward and reliable. In pregnant women, significant changes in waist and hip circumferences during gestation limit their utility as indicators of obesity. However, NC remains relatively stable throughout pregnancy. Similarly, in patients with ascites, the accuracy of waist circumference measurements is compromised, whereas NC remains a stable and reliable metric. In summary, when compared with other anthropometric indices, such as BMI, waist circumference, and hip circumference, NC offers distinct advantages, including simplicity, high reproducibility, and accuracy. Moreover, it remains influenced by factors such as food intake, body position, or respiratory patterns (9, 10).

Prospective cohort studies have shown significant differences in the incidence of non-fatal cardiovascular events across varying NC categories, with event rates of 14.08% in the low, 16.65% in the medium, and 25.21% in the high NC groups (*p <*0.001) (31). These findings are entirely consistent with those observed in our study. International cross-sectional research identified NC as a practical and reliable predictor of CHD (32). Moreover, previous studies have demonstrated that NC serves as an independent risk marker for CHD, surpassing traditional measures such as BMI and waist circumference, with its significance persisting even after adjusting for age and other potential cardiovascular confounders (31, 33, 34). Furthermore, Zen et al. observed that increased adiposity in the cervical region of obese individuals leads to an elevated NC, which is markedly associated with the severity of CHD (35), and similar results were observed in our study. Our analysis revealed that the prevalence of multi-vessel CHD lesions increased progressively with ascending quartiles of NC, compared with the lowest quartile. These findings emphasize the robust association between NC and cardiovascular risk, highlighting its potential as an emerging biomarker for predicting cardiovascular events.

The AIP, recognized for its simplicity and accessibility, has emerged as a potent lipid marker, exhibiting a superior predictive capacity for CHD compared to conventional lipid metrics in recent studies (36, 37). AIP provides a reliable estimate of sdLDL-C (38), which is more prone to infiltrating the arterial wall and forming deposits than LDL-C. Furthermore, sdLDL-C is more susceptible to oxidation, generating oxidized LDL-C, a key contributor to the initiation and progression of atherosclerosis (39). A meta-analysis has demonstrated a significant correlation between elevated AIP levels and a higher prevalence of CHD (12). In a multivariate-adjusted regression model, the risk of CHD progressively increased across higher AIP quartiles compared to the lowest quartile: Q2 (OR: 1.98, 95% CI: 1.52-2.58), Q3 (OR: 3.00, 95% CI: 2.33-3.88), and Q4 (OR: 3.19, 95% CI: 2.47-4.11), with a significant trend observed (*p*-trend < 0.001) (40). Similar results were observed in our study. We found a strong positive relationship between AIP levels and the extent of coronary artery lesions, consistent with the majority of existing literature (41, 42). Additionally, a large-scale prospective cohort study in an international population demonstrated that AIP is significantly associated with cardiovascular disease risk and may serve as an effective tool for mass screening to identify individuals at elevated risk of cardiovascular events (43). In conclusion, AIP may be a promising biomarker for the early detection of CHD, particularly in developing countries (44).

To the best of our knowledge, NC and AIP have emerged as novel biomarkers, distinguished by their ease of measurement, cost-effectiveness, and high patient compliance with periodic assessments. Additionally, the simplicity of these tests enables their widespread implementation across diverse healthcare settings and geographical regions. In the current study, we report a novel linear and positive correlation between NC and CHD for the first time. Moreover, we employed multivariate logistic regression analysis to develop a nomogram that integrated key factors relevant to CHD, thereby identifying the most significant risk factors associated with CHD. This predictive model offers a valuable tool for accurate CHD risk assessment and provides a theoretical foundation for the clinical application of NC and AIP in the identifying high-risk populations for CHD.

In the predictive model, each patient presenting with chest pain was assigned a personalized risk score derived from the nomogram, enabling precise stratification into low- and high-risk groups. When the nomogram indicates a high probability of CHD, further CAG is recommended to identify patients with underlying CHD, given the well-established benefits of timely revascularization. This approach has the potential to substantially enhance patient care by reducing unnecessary invasive procedures and facilitating the timely initiation of appropriate treatments. In future studies, we plan to expand the sample size and perform external validation to further assess the model’s reliability and generalizability. This research provides a foundation for the development of CHD risk prediction tools, software, or applications for patients with chest pain, offering valuable guidance for clinicians in identifying high-risk CHD patients.

This study has several limitations. First, due to its cross-sectional design, it is unable to establish causal relationships between exposure factors and outcomes, thereby limiting the inferences that can be drawn. Future research should incorporate prospective, long-term follow-up data to more comprehensively evaluate the nomogram’s efficacy and the causal associations among the variables. Second, the nomogram was developed using data from a single center, necessitating external validation to ensure its generalizability. Third, although multivariate logistic regression analysis was employed to adjust for potential confounders, the influence of unmeasured confounding factors on the results cannot be excluded. Fourth, the study population consisted solely of hospitalized patients, which may limit the applicability of our findings to outpatient populations. Fifth, while NC measurements were performed by specially trained personnel, potential measurement errors may arise from instrument accuracy, operator proficiency, and the degree of force exerted in the measurement process. Finally, our analysis was confined to participants who underwent CAG, rather than all individuals with suspected CHD, which could potentially introduce selection bias into our results. In summary, further prospective multicenter studies are needed to clarify the complex correlation between NC, AIP, and CHD, thereby providing a more robust scientific foundation for the prevention and treatment of CHD in China.

## 5 Conclusion

In summary, we observed a significant linear and positive correlation between NC, AIP, and CHD. Several critical risk factors for CHD were identified, and a nomogram model was developed to estimate the probability of CHD in patients presenting with chest pain. The combination of NC and AIP demonstrated notable predictive value in diagnosing CHD. However, given the limitations inherent in our cross-sectional study, which limit the ability to establish causality, we emphasize the need for future prospective studies to confirm the causal relationships between NC, AIP, and CHD.

## Data Availability

The raw data supporting the conclusions of this article will be made available by the authors, without undue reservation.
