# Supplementary material for: The correlation between neck circumference and atherogenic index of plasma with coronary heart disease
Source: Front Endocrinol (Lausanne). 2025 Oct 3;16:1562959. doi: 10.3389/fendo.2025.1562959 (PMC12531039; doi:10.3389/fendo.2025.1562959)
Supplement: Supplementary file 1 [file Table1.docx]

***Supplemental Material***

| **Table 1S.** Logistic regression analysis on the association between NC and the severity of coronary lesions | | | | | | |
| --- | --- | --- | --- | --- | --- | --- |
|  | Non-adjusted model | | | Model I | | |
|  | OR | 95% CI | *p-*value | OR | 95% CI | *p-*value |
| NC | 1.05 | 1.01-1.09 | 0.008 | 1.04 | 1.00-1.08 | 0.040 |
| Q1 | Reference | | | Reference | | |
| Q2 | 1.28 | 0.92-1.77 | 0.141 | 1.22 | 0.89-1.65 | 0.213 |
| Q3 | 1.52 | 1.10-2.10 | 0.012 | 1.38 | 1.00-1.90 | 0.050 |
| Q4 | 1.51 | 1.11-2.04 | 0.008 | 1.41 | 1.02-1.95 | 0.040 |
| *p-*trend |  |  | < 0.001 |  |  | 0.039 |

Data are presented as OR and 95% CI. Model I was adjusted for age, gender. OR, odds ratio; CI, confidence interval; NC, neck circumference; Q1, 1st quartile; Q2, 2nd quartile; Q3, 3rd quartile; Q4, 4th quartile.

| **Table 2S.** Logistic regression analysis on the association between NC and the multi-vessel coronary disease | | | | | | |
| --- | --- | --- | --- | --- | --- | --- |
|  | Non-adjusted model | | | Model I | | |
|  | OR | 95% CI | *p-*value | OR | 95% CI | *p-*value |
| NC | 1.04 | 1.00-1.08 | 0.027 | 1.05 | 1.01-1.09 | 0.007 |
| Q1 | Reference | | | Reference | | |
| Q2 | 1.44 | 1.04-2.01 | 0.028 | 1.42 | 1.05-1.91 | 0.023 |
| Q3 | 1.39 | 1.00-1.92 | 0.048 | 1.43 | 1.04-1.96 | 0.028 |
| Q4 | 1.51 | 1.12-2.05 | 0.008 | 1.65 | 1.19-2.27 | 0.002 |
| *p-*trend |  |  | < 0.001 |  |  | 0.005 |

Data are presented as OR and 95% CI. Model I was adjusted for age, gender. OR, odds ratio; CI, confidence interval; NC, neck circumference; Q1, 1st quartile; Q2, 2nd quartile; Q3, 3rd quartile; Q4, 4th quartile.
